# Facilitating Communication With Children and Young Adults With Special Health Care Needs Through a Web-Based Application: Qualitative Descriptive Study

**DOI:** 10.2196/76512

**Published:** 2026-01-06

**Authors:** Jessica R Hanks, Ashley M Hughes, Safura Sultana, Ryan Klute, Kyle Formella, Connor Flynn, Allison Wallenfang, Divya Krishnakumar, Masah Mourad, Yoonje Cho Morse, Matthew J Mischler

**Affiliations:** 1 Department of Health Sciences Education and Pathology College of Medicine Peoria University of Illinois Chicago Peoria, IL United States; 2 Department of Medicine at Metrohealth School of Medicine Case Western Reserve University Cleveland, OH United States; 3 Healthcare Analytics OSF Peoria, IL United States; 4 Innovation OSF Peoria, IL United States; 5 College of Medicine Peoria University of Illinois Chicago Peoria, IL United States; 6 College of Medicine University of Illinois Chicago Chicago, IL United States; 7 School of Public Health University of Illinois Chicago Chicago, IL United States; 8 Department of Medicine College of Medicine Peoria University of Illinois Chicago Peoria, IL United States; 9 OSF Saint Francis Medical Center Peoria, IL United States

**Keywords:** pediatrics, complex care, human-centered design, qualitative, communication

## Abstract

**Background:**

Children and young adults with special health care needs comprise a significant portion of the pediatric population in the United States, where 1 in every 5 children has a complex health care need. These patients are more likely to receive unsafe care and have their needs unmet in part due to lack of accessible information and limited training support. Barriers in communication may contribute to detrimental outcomes for this vulnerable, high-risk population.

**Objective:**

This project aims to identify barriers to communication in children and young adults with special health care needs in the health care setting. These barriers will inform prototype development using human-centered design approaches to create a web-based application. Feedback from patients, caregivers, and health care providers (HCPs) was obtained on the usability and usefulness of the tool within the health care setting.

**Methods:**

A needs assessment was conducted in which participants shared their experiences in providing or receiving health care services via a semistructured interview that was recorded and transcribed. Transcripts were analyzed inductively for themes, coded, and used to categorize the data. On the basis of these themes, iterative development of a web-based application for social stories took place. Focus groups were held to provide relevant feedback on the prototype.

**Results:**

There were 15 participants (n=10, 67% HCPs and n=5, 33% patients and caregivers) interviewed for the needs assessment that informed prototype development. A web-based application for social stories depicting different aspects of health care interactions was created. Focus group feedback from 19 participants (n=12, 63% HCPs and n=7, 37% patients and caregivers) on usability through the System Usability Scale, along with narrative feedback, was obtained. Overall, the usability of the application was supported by caregivers and HCPs.

**Conclusions:**

Children and young adults with special health care needs require medical services that their peers generally do not, thereby compounding potential barriers in communication surrounding health care delivery. Using social stories geared toward health care interactions may help reduce anxiety and difficulty.

## Introduction

Complex care is defined as “a person-centered approach to address the needs of people whose combinations of medical, behavioral health, and social challenges result in extreme patterns of healthcare utilization and cost” [1]. Approximately 20% of adolescents (aged 12-17 years) in North America live with at least one chronic condition or special health care need, >90% of whom will require ongoing care into adulthood [1-3]. Unfortunately, 67% to 75% of individuals living with special health care needs experience frequent visits to the emergency room, forego recommended care (including lack of annual checkups), and frequently have multiple comorbid health issues [2-4]. As a result, their health care expenditures are estimated to be 5 times greater than those of the general population [5,6]. In addition, patients with complex, special health care needs often require individualized treatment plans to overcome the unique barriers they face in obtaining care [7]. These barriers often result in inequities in patient safety and health care outcomes in this population, particularly in individuals with intellectual disabilities [8]. The complexity of navigating health care systems may be lessened with care coordination in a medical home model; there have been reports of improved family satisfaction with overall care and improved health outcomes with dedicated care coordination [9]. As a result, high-quality and timely access to care services [10] and care coordination is cited as a top priority for individuals with disabilities living with specialized health care needs [11].

Complex care approaches focus on the patient, treating them as an individual embedded within a social context. Complex care programs benefit greatly from strong patient–health care provider (HCP) relationships, excellent communication practices, time, and use of interdisciplinary teams who work with specialized care providers to coordinate and provide patient-centered care [12].

Social stories are personalized narratives used to help teach children and young adults with autism spectrum disorder how to navigate social situations [13]. To do this, social stories use a combination of visual aids and text to teach social skills and increase understanding of social context and cues. The structure and predictability of a social story can decrease anxiety in new or unexpected environments. In addition, they can increase a patient’s independence [14] and communication skills, and the skills taught via social stories may then generalize to other social contexts [15].

Patients with complex, special health care needs face several barriers in the health care system when seeking care. These barriers include difficulty communicating with HCPs, a lack of processes to accommodate individual needs, and difficulty accessing recommended care [16]. Specifically for individuals with autism spectrum disorder, patient behavior in combination with deficits in expressive and receptive communication may contribute to challenging medical encounters. A combination of environmental challenges in the setting where medical care is provided, demands placed on the patient (physical examinations or procedure based), and challenges with HCP communication and interaction may invoke challenging behavior in a patient population with reduced communication ability [17].

We sought to address some of these barriers within the health care system for this complex population of patients using the interface between technology and communication. We engaged in a multiphasic study through which we developed an application to address the information and design needs of patients; caregivers; and HCPs who engage in the direct care of patients living with complex, specialized health care needs. The widespread use of technology provides a digital space to create social stories describing health care interactions through visual and narrative means. This intersection of social context in the health care setting may help reduce communication barriers with children and young adults with special health care needs and their caregivers.

We hypothesize that (1) a web-based application can be developed to facilitate communication in the context of health care–specific interactions and flexibility to customize the technology for the patient and (2) a customized web-based application can help close the communication gap that exists for patients with complex, special health care needs, which will lead to fewer poor experiences for patients and caregivers.

## Methods

### Study Design and Setting

We conducted a human-centered design study in three phases consisting of (1) identifying end user needs using qualitative interviews; (2) conducting rapid, iterative prototyping of a web-based application; and (3) holding focus group sessions with end users for prototype feedback. This study relied on a convenience sample recruited from a large academic-affiliated health system (from both outpatient clinic and inpatient units) located in the Midwest region of the United States. The study population included patients with special health care needs, their caregivers, and HCPs who provide health care to patients with special health care needs.

### Ethical Considerations

This project was approved by the University of Illinois College of Medicine Peoria Institutional Review Board under expedited review (2053251). Assent for pediatric patients aged <18 years or adults without decision-making capacity was obtained along with parent or guardian consent. In addition, adults with decision-making capacity provided consent. Transcripts from interviews and focus groups were deidentified before analysis. Participants in the study were not provided with compensation for taking part.

### Human-Centered Design Approach

The first phase of this study comprised semistructured interviews. The interviews were designed to elicit participants’ perceptions of end user communication needs to inform the development of a prototype of a web-based application to facilitate communication among patients, caregivers, and health care personnel. We conducted qualitative interviews to inform the development of a prototype, which was subsequently tested via focus groups with anticipated end users.

### Inclusion Criteria and Participant Eligibility

We gathered a convenience sample from a tertiary care hospital and outpatient care clinic that provides care to patients with special health care needs. To be eligible to take part in all phases of the study, participants needed to meet one of the following criteria: (1) being employed as health care personnel participating in the care of patients with special health care needs in the inpatient or outpatient setting, (2) being a pediatric or adult patient with special health care needs with decision-making capacity who presented for inpatient or outpatient care, or (3) being a caregiver of children or adults with special health care needs who presented for outpatient or inpatient visits.

### Recruitment

Recruitment for participation in the interviews to aid in the development of the web-based application and for the focus groups to provide feedback on the prototype took place via informational study fliers that were distributed electronically via email and physically within inpatient and outpatient clinical settings. The flier included a QR code to a participation questionnaire, as well as QR codes to the various types of age-appropriate consent information, giving prospective participants an opportunity to take part in the study. The participants provided their email address to be contacted for the needs assessment interview via phone call or audio-only Zoom videoconference (Zoom Video Communications).

HCPs providing inpatient or outpatient care were identified by their respective departments and emailed the study details and invitation to participate, in addition to the posted fliers.

Caregivers and patients were primarily recruited using fliers posted in clinical settings. To maximize the likelihood of reaching saturation, patients and caregivers were further identified through convenience sampling. They were contacted via phone or email directly for recruitment. For the purposes of saturation in qualitative methods, we targeted a minimum of 5 participants per category (HCPs, caregivers, and patients) [18].

**Figure 1:**
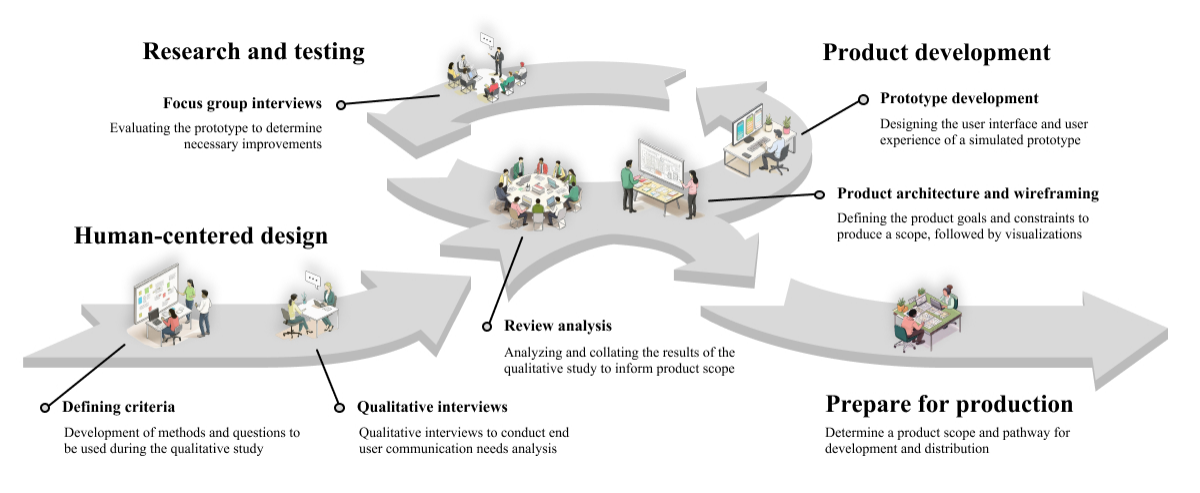
Process map outlining the 3 phases of the study protocol. Phase 1 provided an overview of the human-centered design approach through qualitative interviews, phase 2 incorporated thematic feedback for prototype development, and phase 3 included focus group interviews on the prototype.

After completing the initial interview, participants were asked whether they would like to take part in future focus groups providing feedback on the web-based application prototype (Figure 1).

### Interview Guide Development

The semistructured interview guide underwent several iterations. First, a draft interview guide was developed based on the study aims and clinical context. This was done via discussion among the investigative team; the initial guide was then pilot-tested with 1 caregiver and 1 HCP and subsequently revised. After 4 interviews, transcripts were reviewed in relation to the study’s overarching research question. It was determined that the interview questions were too broad in relation to the study’s research questions, and the guide underwent subsequent revision. Upon approval of the revised questions, follow-up interviews using the new guide were conducted with 2 of the previous 4 participants.

Before conducting interviews, all interviewers underwent interview training. The training materials were developed based on content made available by the University of Illinois Chicago’s School of Public Health Collaboratory for Health Justice [19]. Training materials were presented, followed by opportunities to practice using the guide. The first 2 to 4 interviews were conducted under the supervision of a faculty member with expertise in qualitative human-centered design methods (AMH). Interviews were recorded and transcribed verbatim using Zoom (audio only). A member of the research team reviewed the auto-generated Zoom transcriptions for accuracy and to strip transcripts of identifiable information before analysis.

### Qualitative Analysis

Deidentified transcripts were uploaded to ATLAS.ti (Scientific Software Development GmbH) [20] for subsequent analysis. Approaches to coding were inductive. Coders with backgrounds in medicine (DK), premedicine (MM), and human factors (AMH) reviewed the first 5 transcripts to identify codes and develop a codebook [21]. The remaining transcripts were coded independently by at least 2 trained coders; all discrepancies were identified and resolved for 100% consensus. Emergent themes were identified, sorted into challenges and opportunities, and interpreted for design recommendations. Distributed cognition and communication theories that consider complex interdependencies among multiple individuals, coordinating mechanisms, communication, and information sharing patterns were used for interpretation [22].

**Figure 2:**
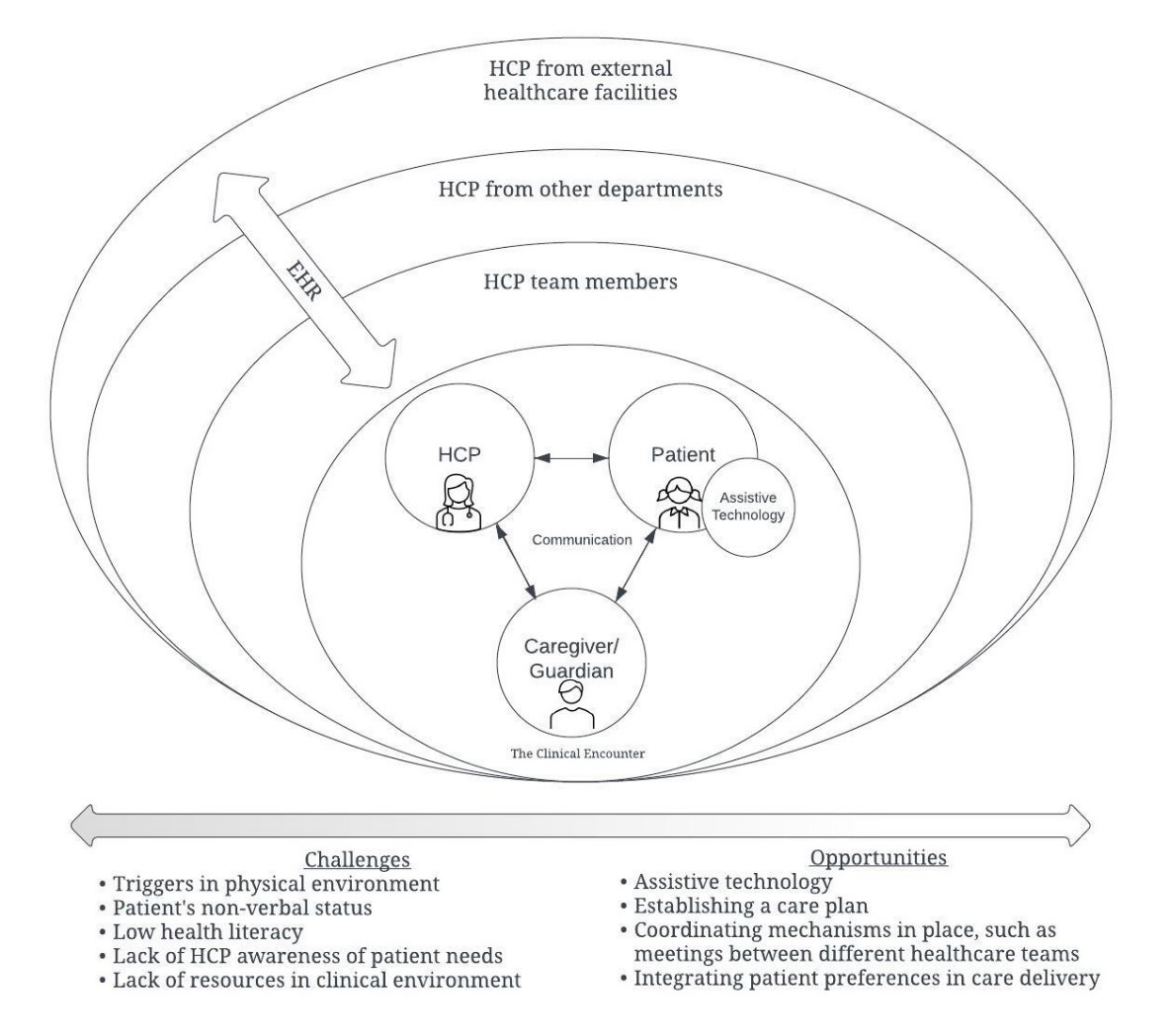
Phase 1 interviews identified opportunities and challenges in communication with health care providers (HCPs) during health care interactions. There are many layers of communication that occur in the coordination of the care of complex patients and their families.

The results identified layers of the health system reliant on communication and coordination practices (Figure 2); challenges and opportunities arise within each layer of the complex system. However, at the core of these interactions is communication, which occurs within the clinical encounter. We thereby focused on codes and emergent themes that attested to challenges and opportunities regarding communication as identified in the clinical encounter.

### Product Development

#### Prototyping

In phase 2, qualitative themes from the interviews were provided to the design team responsible for the development of a prototype for a web-based application through a series of meetings with members of the investigative team (AMH, JRH, and MJM) and designer (KF) to develop multiple iterations of the application. Iterative feedback was received from the investigative team and qualitative lead at that point for clinical expertise (JRH and MJM) and participant voices (AMH) and was incorporated. Each iteration of the prototype was shared with the research team to meet the specifications of the prospectively identified communication and end user needs. Approximately 3 iterative cycles were completed to achieve a final prototype.

#### Clinical Scenario Development

Interviews revealed a common need in both inpatient and outpatient settings: to convey routine tests and procedures in an understandable and nonthreatening manner. To this end, members of the study team developed 2 clinical scenarios through which interaction with the application would guide and convey the steps of a visit or procedure. Our principal investigators, subject matter experts, and internal medicine/pediatric physicians (JRH and MJM) worked closely with content creators to develop a realistic clinical scenario that met the identified areas of need and preference relevant to both inpatient and outpatient care settings. This was developed and refined over several iterations and was ultimately used to inform the flow of the application.

#### Product Design Integration

A user flow was built to represent high-level user experience. An information flow diagram helped identify how this application might connect to other utilities in the health system. A technical assessment began to explore the feasibility of certain features. On the basis of the feedback received, the user flow and technical assessment were refined to address any identified issues and further focus the scope of the application. In conjunction with clinical scenario development, wireframes were built to visualize the prototype layout and structure and develop a preliminary art style for the content. As a result of project team feedback, the wireframes were revised to represent a final scope for the prototype to be used in focus group user testing. Art styles were explored considering the original research data, market evaluation, and potential full-scale production technical constraints. The final phase involved iterating on the art style and developing all necessary design assets to produce a prototype for user testing.

#### Research and Prototype Testing

The third phase of our human-centered design study tested the resulting prototype with end user groups. These user groups consisted of patients, caregivers, and HCPs. For participants aged <18 years, caregivers provided verbal consent, and the participating children provided assent. Decisions to participate were audio recorded. Caregivers of adult patients without decision-making capacity provided consent for the patient to participate.

HCPs, patients, and caregivers who consented to participate convened in person and met in separate focus groups (ie, HCPs met separately from patients and caregivers). Focus group participants were welcomed, provided with the project aims and objectives, provided with a tablet and QR code, and briefed on how to access the prototype application from the tablet provided to them. To interact with the prototype, patients and caregivers were given a medical social story to simulate receiving the social story before an upcoming appointment. They then chose whether they wanted to be represented by an animal or person avatar throughout the medical social story.

HCPs were given the same medical social story under the pretense of having to prepare and send the social stories before an upcoming medical encounter with a patient with special health care needs. All participants were presented with the System Usability Scale (SUS) questionnaire upon completion of the scenarios and then took part in a facilitated focus group interview session to provide more granular feedback. Focus group interviews were recorded and transcribed verbatim with participant consent.

### Focus Group Data Collection Forms

To evaluate prototype success in the focus group, we adapted survey questions from the SUS [23] and from existing educational immersion scales [24]. Furthermore, we used a semistructured guide to elicit open-ended feedback on the application experience from end users. The SUS is a scale designed to measure participants’ perceived usability of a product. Scores range from 0 to 100, with the average score (50th percentile) being 68 [25]. A score above 70 is generally considered acceptable [26-28]. The Learning Immersion Scale in Simulation is a psychometrically validated and reliable survey consisting of 4 factors: cognitive assimilation, emotional buy-in, focused attention, and autotelic experience [24]. We focused on the cognitive assimilation subscale, which measures to what extent an individual differentiates between interaction with the simulated environment and reality [29]. The questionnaire uses a 7-question, 5-point scale adapted from Ko et al [24]. It ranges from “strongly disagree” (1) to “strongly agree” (5). This scale was only administered to caregivers and patients.

### Focus Group Data Analysis

SUS surveys were pooled and analyzed descriptively, examining overall usability and usability differences by role (caregivers and patients vs HCPs). The caregiver or parent survey asked 3 additional questions about how they thought their child or the individual under their care would feel about the prototype, which caused the SUS scale score range to change to 0 to 130. Scores are reported as both out of 130 and normalized to fit the original scale from 0 to 100*.* The resulting focus group transcripts were reviewed iteratively for trends, including areas of feedback (positive and negative) on the prototype interaction experience.

## Results

### Overview

Results are presented and discussed in the order in which the phase of research took place. There were 15 participants (n=10, 67% HCPs and n=5, 33% patients and caregivers) in the phase 1 individual interviews (Table 1).

**Table 1:** Participant demographics.

	Patients and caregivers, n (%)	Health care providers, n (%)
Phase 1 interviews (n=15)	5 (33)	10 (67)
Phase 3 focus groups recruited in phase 1 (n=9)	4 (44)	5 (56)
Phase 3 focus groups recruited in phase 3 (n=10)	3 (30)^a^	7 (70)
Phase 3 total (n=19)	7 (37)	12 (63)

^a^One pediatric participant.

### Human-Centered Design

Qualitative analysis of the interviews completed in phase 1 identified both challenges and opportunities of care, illustrating design needs for our web-based application. Themes were identified to help prioritize the creation of a web-based application as a potential solution to reduce challenges and facilitate interaction (Multimedia Appendix 1).

#### Challenges in Communication Based on Cognitive Status

Equating verbal status with cognitive ability emerged as a barrier to effective communication. This challenge was mentioned by HCPs, with 1 patient and 1 caregiver noting this barrier from their perspective. Mainly, HCPs indicated difficulty assessing patient capabilities and capacity to communicate and understand interactions autonomously due to time constraints or unfamiliarity with the patient:

Because a lot of times they are so afraid of what’s going on, and they’re not understanding. And sometimes...providers in the room aren’t understanding them. And that can cause a lot of problems.

HCP

That they’re special needs and they don’t have the capacity when so many of them have the capacity we [HCPs] just don’t have the time to spend with them to understand what they do have.

HCP

Meanwhile, patients and caregivers described how this challenge manifested on their end in that HCPs may not provide enough information on what they do with a patient, expressing a desire for more explanation that warranted more communication. One caregiver described their intensive care unit experience as follows:

[A] nurse will come in and start something [a routine medical procedure]. You’re like, wait a minute, what are you doing? So I don’t know if like just a little more communication as to the doctors thought processes of: This is what we’re doing. This is what we’re thinking and what we’re going to try.

Caregiver

Several caregivers noted that the barrier was the verbal status (eg, the patient’s ability to talk) rather than their cognitive ability or capacity for understanding. One caregiver noted the following:

Just because he can’t talk doesn’t mean he doesn’t have feelings and doesn’t understand everything.

Caregiver

Now his mom never left she was able to communicate a lot with us [HCP team].... I didn’t know him [the patient] very well. And so if mom worked or couldn’t be here, there would have been a lot of gap(s) in communication and understanding what he needed.

HCP

Some HCPs corroborated this element of patient understanding despite verbal status:

[Patient name] is very, very smart and understands a lot of what we are saying or doing. So even though a child may have special needs and are non-verbal and not able to communicate what they want, how we do, I feel like it's important to know that they still sometimes are aware of their surroundings and are smarter than we really realize.

HCP

#### Opportunities to Improve Communication

The challenge of ascertaining a patient’s cognitive status is prevalent, often requiring intervention and the constant presence of a caregiver. Overall, there was a preference for more streamlined and direct communication between HCPs and patients. Ideas for supporting direct communication during an encounter included the use of simplified messaging. For instance, one HCP highlighted the need to use simple language to explain routine procedures in terms readily understood by patients and caregivers:

Yes, and it fits people that are aware of medical jargon...[but] our special needs are not. And so we have to be able to. Adjust [using jargon] as according to our patients you know.

HCPs

Assistive devices were noted in their ability to promote more direct and patient-centered communication during clinical encounters, potentially bypassing the otherwise constant need for caregiver presence. These devices were often noted to be available through specialty hospital services or otherwise belong to the patient privately for at-home use, and it was noted that they were useful in the health care environment to aid in direct communication between HCPs and their patients. In other cases, involving the caregivers in the health care delivery provides insights into the patient’s mannerisms and needs in a way that only those who know them best can decipher. One HCP noted the following:

[D]epending on what’s going on with them. Absolutely. Yeah, so more individuality in a system, you know. Ability to change it to a specific patient would be helpful.

HCP

So if they are like not actually having access to smart tablets or for understanding and navigating the My Chart system. So that technology.... To feel comfortable with doing that is one of the barriers.

HCP

When describing aspects of an assistive device used for communication, several facilitating device features were noted to enhance communication during the clinical encounter:

So from, provider to patient communication, just having some, a bunch of preset sort of procedures, the very, you know, illustrations that sort of simplify it and make it easy to understand for the patient and then on the opposite, you know, things that allowed them to quickly say and communicate with you without.... That doesn't mean they're the same as everyone else. And so I think a goal with the app is to sort of a breakdown communication barriers so that there’s an easier time getting to know them and then also you know those individual preferences as individual traits and things like that can be quickly communicated and just sort of be embedded and quickly understood by providers.

HCP

### Prototype Development

In the process of prototype development in phase 2, we focused on actionable themes that met the following criteria: (1) opportunities to improve communication in clinical encounters (the reason for this being that broader coordination issues worsened in part because of communication problems, ie, communication is often necessary for coordination) and (2) common or routine tests and procedures experienced in both inpatient and outpatient encounters.

A tappable prototype was built and prepared for testing on tablet devices. Story development and key features targeted emergent qualitative themes, with a focus on routine procedures performed in both inpatient and outpatient visits and on a standardized outpatient visit.

Common procedures patients undergo in both settings are phlebotomy and measurement of vital signs, and a common visit focus in the outpatient setting is a preventative examination or annual physical examination (including obtaining vital signs). An outline of narrative details was created that would help shape the final visual representation of each step within the procedure or visit. A landing page of potential scenarios for social stories was created (Figures S1 and S2 in Multimedia Appendix 2), with the standardized outpatient visit fully developed with 2 different representations, an animal or a person avatar. This allowed the end user to choose the avatar that best reflected their desire to represent themselves in an upcoming encounter.

### Research and Prototype Testing

The scope of the themes was then identified by the research team to provide the framework for the iterative prototyping of the web-based application. The web-based application focused on providing a visual and narrative aid to explain treatments or procedures to the patients and caregivers. It also provided the opportunity for the patient to react to the content (Table S1 and Figure S1 in Multimedia Appendix 3).

**Figure 3:**
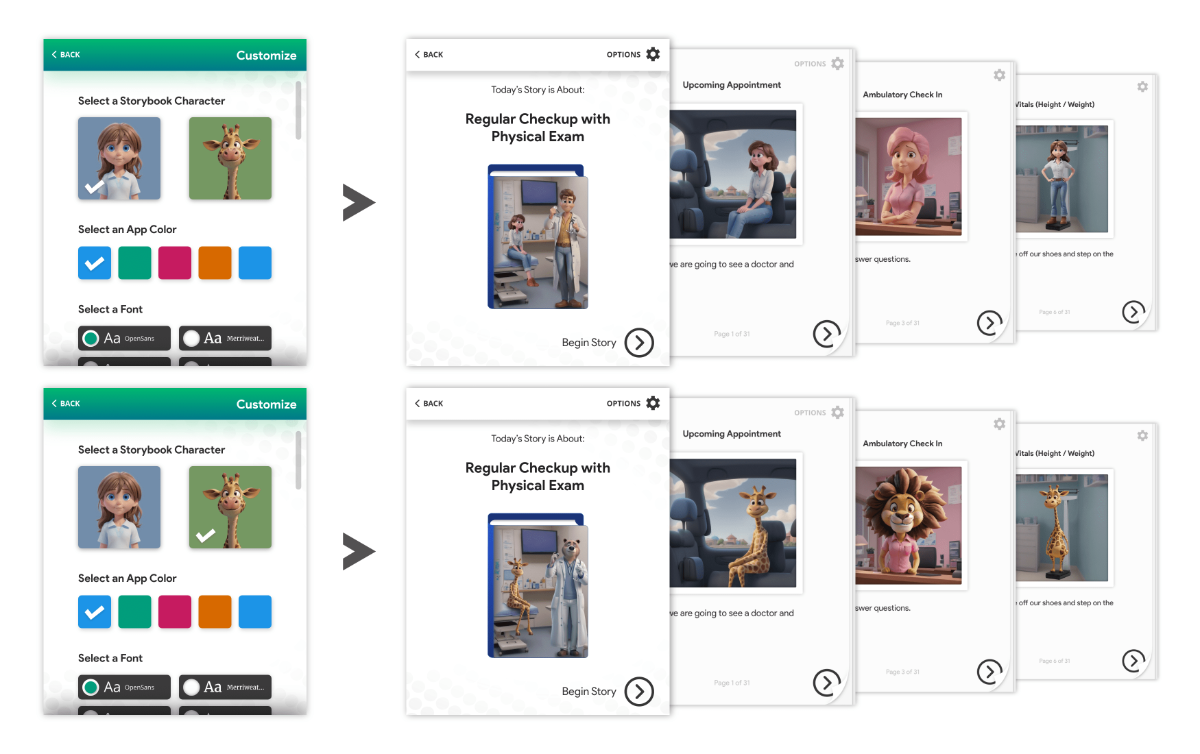
Social story outlining the steps of a routine outpatient preventative examination. The patient or caregiver can select between an animal and a person avatar that represents them throughout the social story.

In the phase 3 focus groups, there were a total of 19 participants (Table 1) who provided feedback on the visual and narrative examples (Figure 3) for the web-based application.

### SUS Results

#### HCP Results

On the basis of the HCP SUS scores (Table 2), the prototype ranked in the “best imaginable” range with an average score of 93.125 (SD 6.67; 96th-100th percentile). However, due to the small sample size, more participants would be needed to accurately judge the usability and generalizability of the prototype.

**Table 2:** System Usability Scale scores from health care providers.

Respondent ID	Score (range 0-100)^a^
HCP1	97.5
HCP2	100
HCP3	90
HCP4	92.5
HCP5	100
HCP6	90
HCP7	97.5
HCP8	100
HCP9	97.5
HCP10	87.5
HCP11	80
HCP12	85

^a^Average score: 93.125 (SD 6.67).

#### Caregiver Results

The caregiver SUS scores (Table 3) also ranked the prototype in the “best imaginable” range, with an average normalized score of 88.08 (96th-100th percentile).

**Table 3:** System Usability Scale scores from caregivers.

Respondent ID	Original score (0-130)^a^	Normalized score (range 0-100)^b^
CGP1	127.5	98.08
CGP2	112.5	86.54
CGP3	97.5	75
CGP4	125	96.15
CGP5	110	84.62

^a^Average score: 114.5 (SD 12.17).

^b^Average score: 88.08 (SD 9.36).

#### Patient Results

The 2 patients surveyed rated the prototype just below the average SUS score with an average score of 66.25 (SD19.45; 41st-59th percentile). This would place it in the “OK” range.

### Cognitive Assimilation

Caregivers (n=5) consistently provided high scores on the cognitive assimilation scale, with the lowest score being 4.2 for “I was able to see it I was doing it right” and “The situation seemed to flow smoothly” (Table S1 in Multimedia Appendix 4). However, patients (n=2) provided low scores on cognitive assimilation, with an average of 2 for most of the questions (Table S2 in Multimedia Appendix 4).

### Focus Group Themes

Common themes from both HCPs and patients and caregivers on the prototype centered on the user-friendly application design, including the avatar choice between an animal and a person. The design was overall appreciated as a general medical social story. However, broad use among patients with special health care needs of all age ranges may be limited by the spectrum of ability. The current narrative explanations and visual aids are most applicable to patients of a certain cognitive ability regardless of chronological age. When used outside of that scope, they may not be as effective for end user interaction. However, both groups generalized applicability to neurotypical children of a similar cognitive level:

Yeah, I thought that it was really well laid out. I thought that as a mom and then also as like I’m a pediatric nurse. So from both of those aspects, I feel like this is something that we’re currently missing. We’re not able to make those connections with our pediatric patients because you know we don’t have a lot of child friendly resources to kind of help them prepare for different things that they may go through in their health care process.

Caregiver

Both patients and caregivers and HCPs provided feedback on preferences regarding the ability to modify the scope of the medical social story for various end users, including narrative explanations, length, and increased avatar options, which may increase general applicability and end user satisfaction:

You know some more of an adult or approaching adults. A tween? Yeah, a tween. I would expect most children would pick the giraffe. Yeah, but I would think more teens and tweens would choose a teenager type avatar. That would kind of bridge the gap between the very childlike and juvenile appearance and the more capturing the ages in between.

HCP

Caregivers and patients with specific health care needs identified potential areas of improvement to meet broader patient care accessibility needs. The addition of an audio version with sign language should be considered so that individuals that are hard of hearing or cannot read can still use the application fully.

In the HCP feedback specifically, there was some concern about being able to capture the many different permutations through which an HCP could approach a case to meet the needs of each individual patient or account for variances within a health care system in terms of what information is included. In addition, as the nature of patients with special health care needs may result in frequent encounters with the health care system, the application needs to have the ability to be personalized for repeated similar health care interactions for patients:

We use freezy spray so I can speak to that, but you know the hospital will use numbing cream, you know, and it is, you know, you use these words, but they have different things that we can use. Some people use a little light to see your vein. Sometimes you know.

HCP

Going back to this surgery example, kids have to be under a certain weight to get a mask induction versus the IV induction and so you can’t even break it down by 8 years if you have a 7-year-old that weighs the same as in 12-year-old. The seven-year-old will get the IV right? Yeah, it it’s very specific.

HCP

## Discussion

### Principal Findings

The initial objective of this project was to create a web-based application that would facilitate communication for patients with complex, special health care needs during interactions with their health care team. Through a 3-phase human-centered design process, we identified communication needs for patients with complex, special health care needs; their caregivers; and their HCPs. Key themes informed prototype development. The prototype was then reviewed by a group of stakeholders; the results of focus groups with end users support overall usability and utility for certain patient populations.

The results from the phase 1 interviews identified areas of opportunities and challenges in communication for patients with special health care needs in health care encounters. Specifically, HCPs’ level of comfort and inclusive communication with the patients and their caregivers during clinical encounters were two areas identified as needing improvement. Patients notably display a broad range of communication abilities, particularly when using alternative and augmentative communication methods. Interestingly, capacity for direct verbal communication may not directly reflect an individual’s cognitive ability. This finding highlights how assumptions on patient function and ability and possible ableism may create additional communication barriers during a patient encounter. Additionally, a high reliance on caregivers for verbal communication of the patient status may further complicate the patient’s relationship with the health care team, whereas using communication devices available allows for direct communication with the patient. This was consistent with our initial hypothesis and experience within the health care system. Although our data suggest additional multiple areas of opportunity to enhance communication, we focused on the findings most relevant to the interactions among HCPs, patients, and caregivers during an encounter for prototype development. In phase 2, construction of the prototype elements was important to consider for scalability and future digital interfaces. The design of the medical social stories was chosen to depict routine experiences for all patients and their families but potentially significant barriers for patients with complex, special health care needs due to the physical environment and impaired communication and rapport with the health care team. Generated images of an animal or person avatar were incorporated to provide options for individual preferences, but creating avatars using personalized variables was not possible during prototype creation to remain within the scope. Striking an appropriate balance among the development of a web-based application, functionality, and scalability was at the forefront of our decisions when finalizing prototype details. In phase 3, the qualitative feedback from stakeholders highlighted consistencies and potential applicability to a focused patient population with communication barriers and intellectual disabilities. The qualitative feedback from HCPs and caregivers identified that this prototype may be applicable to patients with typical development at the same cognitive level. Overall, caregiver and HCP testing was supportive of the prototype meeting the standards for usability [30]. The combination of the qualitative and quantitative data was positively congruent with HCPs and caregivers, but the small sample size prevents solid conclusions on usability and generalizability to both other institutions and other patient groups with special health care needs.

Limitations of this study include the representativeness of the sample in both size and diversity of disease processes that result in specialized health care needs. Therefore, the full scope of opportunities and challenges faced by patients across the continuum may not be identified. Thus, the broad applicability of a prototype designed for all patients with special health care needs would need further validation across a larger sample size and across multiple areas of care delivery within the health care system. In addition, there was a broad range of levels of ability among patients in the phase 1 interviews, which created variable outcomes in the design of the prototype. Building a prototype that would meet the needs of an innately unique population of patients with varying levels of ability within the same diagnoses exceeded the scope and timeline of the first cycle of this project but is a rich area for further refinement and implementation. Finally, just as the patients are unique, so are the individual approaches that health care team members bring to their patient care. For example, the approach to anesthesia for a surgical procedure may vary depending on the HCP and the patient’s unique needs, which, therefore, could limit the generalization of the medical social story.

### Conclusions

Our study identified an area of further exploration for increasing successful health care interactions with patients with special health care needs through using social stories in a web-based application. Providing a mechanism to prepare patients and their caregivers for health care interactions by introducing a standardized process allows for structure, visibility, and appropriate anticipation. This also allows for the engagement of HCPs to communicate with patients and families before busy encounters to help set expectations and also potentially reduce anxiety about the unknown. As noted in the stakeholder feedback, these features are also applicable for health care interactions involving neurotypical patients. Future areas of research will be to further the development of the web-based application, including expansion of the medical social stories for increased applicability and evaluating implementation in the workplace for feasibility. In future ambulatory workflow states, distributing the relevant social stories through the electronic medical record before an upcoming health care encounter would mimic the use of social stories in other settings. During inpatient admissions, the social stories may be deployed by caregivers or bedside nursing staff for individualized preparation for the health care process and procedures. Once the workflows are established, an evaluation of patient, caregiver, and HCP satisfaction is warranted to ensure usability and impact. In addition, consideration of a built-in functionality for a caregiver or patient to select an estimated cognitive ability, which may increase applicability to broader neurotypical and neurodivergent patient populations, would require flexibility in terms of social story content and avatar selection. Finally, other common themes that were identified as areas of need were the ability to provide an updated interface with the health care team highlighting important, individualized care elements; patient advocacy; and preferred interfaces with electronic health records.

## Appendix

Multimedia Appendix 1 Codes used in the qualitative analysis of phase 1 interviews with patients, caregivers, and health care providers.

Multimedia Appendix 2 Screenshot of the web-based application home page and of the landing page depicting the table of contents of potential social stories for health care interactions.

Multimedia Appendix 3 Outline of the thematic takeaway from phase 1 for the creation of the prototype and potential directions for future iterations and an accompanying visualization of the function of the prototype.

Multimedia Appendix 4 Cognitive assimilation scores for caregivers and patients. Scores measure to what extent an individual differentiates between interaction with the simulated environment and reality.

## Abbreviations

HCP: health care provider
SUS: System Usability Scale

## Data Availability

The datasets generated or analyzed during this study are available from the corresponding author on reasonable request.
