# Supplementary material for: Facilitating Communication With Children and Young Adults With Special Health Care Needs Through a Web-Based Application: Qualitative Descriptive Study
Source: JMIR Pediatr Parent. 2026 Jan 6;9:e76512. doi: 10.2196/76512 (PMC12820544; doi:10.2196/76512)
Supplement: Multimedia Appendix 1 [file pediatrics_v9i1e76512_app1.docx]

Table S1 Multimedia Appendix 1. Codes used in qualitative analysis from phase one interviews with patients, caregivers and healthcare providers.

| Code | Definition | Frequency |
| --- | --- | --- |
| Verbal/Non-Verbal | Need for establishing patient's capacity for communication prior to clinical encounter | 11 |
| Family | Need for establishing the degree of involvement of the family in clinical care | 29 |
|  | Reliance of HCP on family during patient-provider interaction for communication and information |  |
| Barriers to Care Coordination | When problems arise between sharing information (between multiple HCP and/or HCPs and care givers) | 19 |
| Care coordination | Coordination between multiple HCP (often interprofessional) | 22 |
|  | Coordination and information sharing between the caregivers and HCP |  |
| Electronic Health Record | Integration of the EHR into communication (HCP - CG, HCP - HCP) and care plan development (can also be referred to as electronic health record [EHR] or electronic medical record [EMR], the vendor name for the type of record being used at the current health system Epic) | 14 |
| Resources | Inadequate staffing, medical devices, time etc. | 27 |
| Patient Advocacy | taking the initiative to communicate patient needs to HCP (normally when patient needs are not being met) | 15 |
| Barriers to Communication | Aspects of clinical encounters that impair communication | 32 |
| Facilitators to Communication | Aspects of clinical encounters that improve communication | 22 |
| Patient Preferences | Inclusion of information that are may not be clinically relevant, but will improve the patient's experience. Accommodating for patient needs. | 22 |
| Bedside Manner | Actions taken by HCP to facilitate the clinical encounter (e.g. talk before touch) | 15 |
| HCP Experience/Comfort | Whether or not the HCP has had prior experience caring for patients in this population, and how comfortable they appear to be in the situation | 22 |
| Environment | Elements/triggers present in the environment that impact the clinical encounter (e.g. distracting machines) | 10 |
| Social context | Lack of access to resources, ability to access healthcare | 13 |
| Health literacy | Patients/caregiver's ability to understand diagnosis and care needs | 13 |
| Competency | HCP having the appropriate training to handle the situation, taking the appropriate decisions within reasonable time period | 22 |
| Patient Compliance | Capacity of the patient to follow the HCP's directives | 5 |
| Continuity of Care | Consistency in which clinicians are providing care to the patient over time | 11 |
