# Supplementary material for: Facilitating Communication With Children and Young Adults With Special Health Care Needs Through a Web-Based Application: Qualitative Descriptive Study
Source: JMIR Pediatr Parent. 2026 Jan 6;9:e76512. doi: 10.2196/76512 (PMC12820544; doi:10.2196/76512)

Figure S1 Multimedia Appendix 2. Screenshot of web-based application home page.


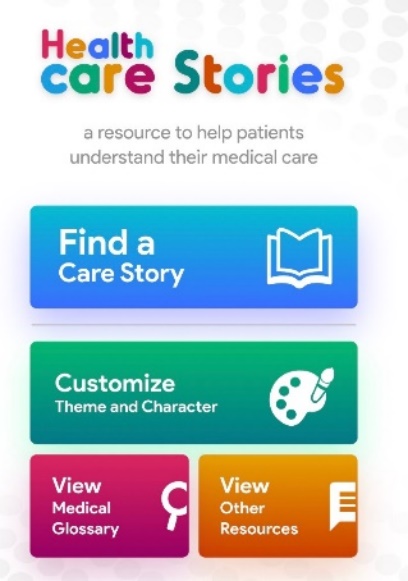


Figure S2 Multimedia Appendix 2. Screenshot of web-based application landing page depicting table of contents of potential social stories for healthcare interactions.


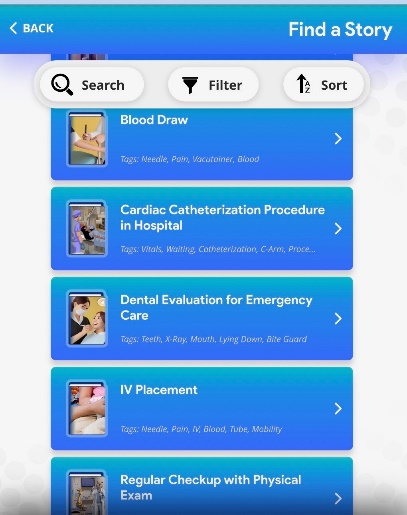

Supplement: Multimedia Appendix 2 [file pediatrics_v9i1e76512_app2.docx]
