# Supplementary material for: Facilitating Communication With Children and Young Adults With Special Health Care Needs Through a Web-Based Application: Qualitative Descriptive Study
Source: JMIR Pediatr Parent. 2026 Jan 6;9:e76512. doi: 10.2196/76512 (PMC12820544; doi:10.2196/76512)
Supplement: Multimedia Appendix 3 [file pediatrics_v9i1e76512_app3.docx]

Table S1 Multimedia Appendix 3. Outline of thematic take away from phase 1 for creation of prototype and potential direction for future iterations.

| Code | Definition | Prototype |
| --- | --- | --- |
| Verbal/Non-Verbal | Need for establishing patient's capacity for communication prior to clinical encounter | Buttons to react to social story |
| Family | Need for establishing the degree of involvement of the family in clinical care | Future state that could be used to communicate plans in absence of caregiver. |
|  | Reliance of HCP on family during patient-provider interaction for communication and information |  |
| Care coordination | Coordination between multiple HCP (often interprofessional) | Future state if chat function or interface with EHR |
|  | Coordination and information sharing between the caregivers and HCP |  |
| Barriers to Communication | Aspects of clinical encounters that impair communication |  |
| Facilitators to Communication | Aspects of clinical encounters that improve communication | Images explaining procedure/visit |
| Patient Preferences | Inclusion of information that are may not be clinically relevant, but will improve the patient's experience. Accommodating for patient needs. | Colorful, pictures  Exciting  Big buttons  Stories |
| HCP Experience/Comfort | Whether or not the HCP has had prior experience caring for patients in this population, and how comfortable they appear to be in the situation | Future state consideration of understanding patient preferences |

Figure S1 Multimedia Appendix 3. Visualization of function of the prototype
