# Supplementary material for: Facilitating Communication With Children and Young Adults With Special Health Care Needs Through a Web-Based Application: Qualitative Descriptive Study
Source: JMIR Pediatr Parent. 2026 Jan 6;9:e76512. doi: 10.2196/76512 (PMC12820544; doi:10.2196/76512)
Supplement: Multimedia Appendix 4 [file pediatrics_v9i1e76512_app4.docx]

Table S1 Multimedia Appendix 4. Cognitive assimilation scores for caregivers. Scores measure to what extent an individual differentiates interaction with the simulated environment and reality.

| **Caregiver**  **Cognitive Assimilation** | |
| --- | --- |
| **Question** | **Average** |
| Flows Smoothly | 4.2 |
| Knew what to do | 5 |
| Information put into one | 4.8 |
| Control | 5 |
| Knowledge came together | 5 |
| Understood flow of scenario | 5 |
| Doing it right | 4.2 |

Table S2 Multimedia Appendix 4. Cognitive assimilation scores for patients.

| **Patient**  **Cognitive Assimilation** | |
| --- | --- |
| **Question** | **Average** |
| Flows Smoothly | 2 |
| Knew what to do | 2 |
| Information put into one | 2 |
| Control | 2 |
| Knowledge came together | 2 |
| Understood flow of scenario | 2.5 |
| Doing it right | 2 |
